# Viruses and Metabolism: The Effects of Viral Infections and Viral Insulins on Host Metabolism

**DOI:** 10.1146/annurev-virology-091919-102416

**Published:** 2021-09-29

**Authors:** Khyati Girdhar, Amaya Powis, Amol Raisingani, Martina Chrudinová, Ruixu Huang, Tu Tran, Kaan Sevgi, Yusuf Dogus Dogru, Emrah Altindis

**Affiliations:** Department of Biology, Boston College, Chestnut Hill, Massachusetts 02467, USA

**Keywords:** viruses, viral insulins, metabolism, glycolysis, lipid metabolism, glutaminolysis

## Abstract

Over the past decades, there have been tremendous efforts to understand the cross-talk between viruses and host metabolism. Several studies have elucidated the mechanisms through which viral infections manipulate metabolic pathways including glucose, fatty acid, protein, and nucleotide metabolism. These pathways are evolutionarily conserved across the tree of life and extremely important for the host’s nutrient utilization and energy production. In this review, we focus on host glucose, glutamine, and fatty acid metabolism and highlight the pathways manipulated by the different classes of viruses to increase their replication. We also explore a new system of viral hormones in which viruses mimic host hormones to manipulate the host endocrine system. We discuss viral insulin/IGF-1-like peptides and their potential effects on host metabolism. Together, these pathogenesis mechanisms targeting cellular signaling pathways create a multidimensional network of interactions between host and viral proteins. Defining and better understanding these mechanisms will help us to develop new therapeutic tools to prevent and treat viral infections.

## 1. INTRODUCTION

Viruses are the most abundant and widespread biological entities in the world with an enormous variety of genetic material and the ability to infect different species (1). As a result of coevolution, viruses have developed various mechanisms to facilitate viral replication. One of these mechanisms is based on manipulating the host metabolism by disrupting critical metabolic pathways and targeting master regulator proteins of metabolism. Metabolic signaling pathways are the main decision-making processes that coordinate cell signaling; gene transcription and precise modulation of these pathways are vital for organisms. Thus, viruses have evolved to target these pathways and alter metabolism. Over the past three decades, several groups have also explored and identified mechanisms exploited by viruses to increase macromolecules including glucose and fatty acid level in the cells for their replication (2–5). In this review, we explore the manipulation of metabolic pathways by different classes of viruses that can infect humans or other distinct animal species. We cover the studies that identified an exact mechanism of viral manipulation of metabolic signaling; however, we exclude the descriptive studies. Successful characterization of these modified signaling pathways has the potential to be the key to the treatment of several viral infections.

## 2. GLUCOSE METABOLISM

**Figure 1:**
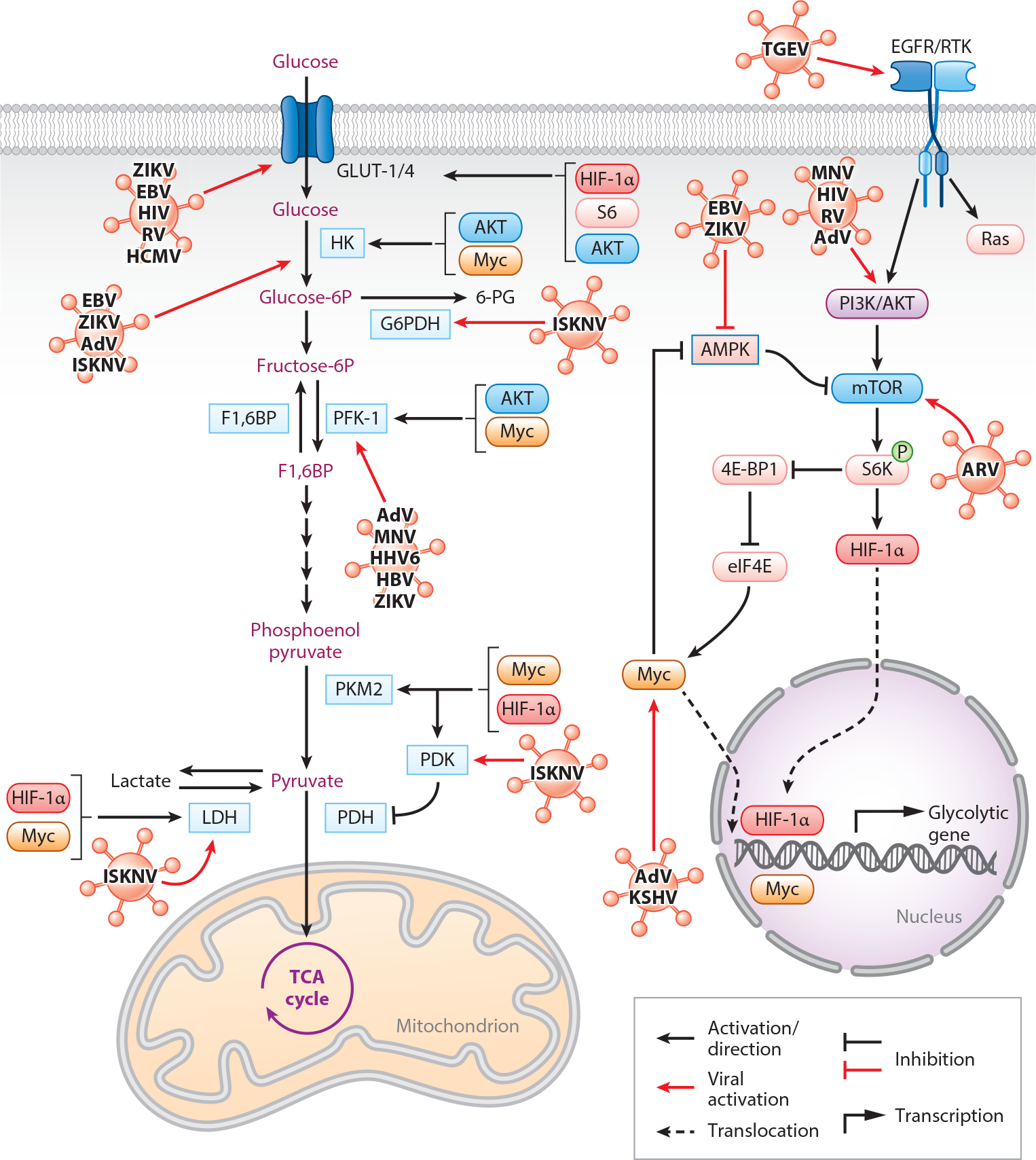
Glucose metabolism and related signaling pathways are altered by viruses. Upon infection, there is an increase in the rate of glycolysis that is mainly accomplished by an increase in GLUT activity, rate-limiting glycolytic enzyme activity, and several signaling proteins and transcription factors. Abbreviations: 4E-BP1, 4E-binding protein 1; 6-PG, 6-phosphogluconate; AdV, adenovirus; AMPK, AMP-activated protein kinase; ARV, avian reovirus; EBV, Epstein-Barr virus; EGFR, epidermal growth factor receptor; eIF4E, eukaryotic translation initiation factor 4E; F1,6BP, fructose-1,6-bisphosphate; G6PDH, glucose-6-phosphate dehydrogenase; GLUT, glucose transporter; HBV, hepatitis B virus; HCMV, human cytomegalovirus; HIF, hypoxia-inducible factor; HIV, human immunodeficiency virus; HK, hexokinase; ISKNV, infectious spleen and kidney necrosis virus; KSHV, Kaposi’s sarcoma herpesvirus; LDH, lactate dehydrogenase; MNV, murine norovirus; mTOR, mechanistic target of rapamycin; Myc, proto-oncogene, basic helix-loop-helix transcription factor; P, phosphate group; PDH, pyruvate dehydrogenase; PDK, pyruvate dehydrogenase kinase; PFK-1, phosphofructokinase 1; PI3K, phosphoinositide 3-kinase; PKM2, pyruvate kinase M2; Ras, rat sarcoma; RTK, receptor tyrosine kinase; RV, rhinovirus; TCA, tricarboxylic acid; TGEV, transmissible gastroenteritis virus; ZIKV, Zika virus. Created with BioRender.com.

Glucose metabolism is a complex process through which cells convert glucose into energy and other metabolic intermediates (Figure 1). The first step of this process is glycolysis, an ancient, evolutionarily conserved, oxygen-independent process with two main functions, the generation of ATP and the generation of pyruvate, both of which are used in the later stages of cellular respiration (6). Following glycolysis, in the presence of oxygen, pyruvate is oxidized into acetyl-CoA and utilized in the tricarboxylic acid (TCA) cycle, also known as the Krebs cycle. The TCA cycle is a series of reactions used to release energy and produce redox cofactors from acetyl-CoA oxidation through a series of redox reactions. Redox cofactors then contribute to oxidative phosphorylation (OXPHOS), where electrons donated from NADH and FADH are shunted through the electron transport chain (ETC), causing the production of a gradient of H^+^ molecules that allows for the generation of ATP, making OXPHOS a significant source of ATP in aerobic organisms (7).

An integrated metabolic signaling network plays a significant role in the regulation of glucose homeostasis by a feedback loop at the level of phosphofructokinase (PFK), the rate-limiting enzyme of glycolysis. In a state of low ATP/AMP, the energy-sensing enzyme AMP-activated protein kinase (AMPK) suppresses energy-consuming pathways. It thus augments glucose uptake to maintain homeostasis and decreases bioenergetics stress (8). Growth factors such as platelet-derived growth factor, erythropoietin, epidermal growth factor (EGF), transforming growth factor, and cytokines stimulate glucose uptake and cell proliferation via activation of the phosphoinositide 3-kinase (PI3K)/protein kinase B [PI3K/PKB(AKT)] pathway. This promotes cell cycle progression and protein translation by activating the mechanistic target of rapamycin (mTOR) protein (9).

Viruses manipulate glucose metabolism to increase available energy and promote their own reproduction by modulating the aforementioned signaling pathways. Most viruses accomplish this by inducing aerobic glycolysis, also known as the Warburg effect (2). The Warburg effect is the fermentation of pyruvate at the end of glycolysis into lactate using lactate dehydrogenase (LDH), even when oxygen is present. Thus, it modulates the key regulatory enzymes of aerobic glycolysis, glucose transporters (GLUTs), hexokinase (HK), PFK, pyruvate kinase, and LDH. The result of aerobic glycolysis is buildup of more lactic acid, fewer glycolytic intermediates contributing to the TCA cycle, and high consumption of glucose (10).

Based on the genome structure [single-stranded or double-stranded (ds) DNA or RNA] and the mode of replication, viruses are categorized into seven classes (I–VII). Different classes of viruses such as Class I white spot syndrome virus (WSSV) (11), human papillomavirus 16 (12), human cytomegalovirus (HCMV) (13), and Kaposi’s sarcoma herpesvirus (KSHV) (14) and Class IV hepatitis C virus (HCV) (15) use various mechanisms to induce this metabolic shift, which is often essential for viral replication (Supplemental Table 1). For example, severe acute respiratory syndrome coronavirus 2 (SARS-CoV-2) (Class IV) infection in humans causes an energy deficit by disrupting mitochondrial function that is then compensated for by increased glycolysis (16). Infectious spleen and kidney necrosis virus (ISKNV) (Class I) that infects fish induces higher expression of glycolytic enzymes HK1, LDH, and glucose-6-phosphate dehydrogenase to provoke its replication in Chinese perch brain cells. Subsequent inhibition of pyruvate dehydrogenase kinase, and thus disruption of aerobic glycolysis, was shown to inhibit ISKNV replication (17).

### 2.1. Signaling Pathways Manipulated by Viruses to Induce Glucose Metabolism

AMPK is a sensor of cellular energy conserved in all eukaryotic cells, making it an important target for several viruses. AMPK signaling inhibits the mTOR responsible for cell growth and proliferation (18). Furthermore, AMPK signaling activates hypoxia-inducible factor-1 (HIF-1), which upregulates glucose uptake and glycolytic enzymes (19). In normal cells, increased AMPK signaling promotes glycolysis and ATP formation. Paradoxically, increased AMPK signaling under cell stress conditions (such as in cancer or viral infection) decreases glycolysis (20). EBV-miR-Bart1-5P, one of the 44 mature microRNAs encoded by Epstein-Barr virus (EBV) (Class I) that infect humans, was shown to promote aerobic glycolysis and induce angiogenesis in nasopharyngeal carcinoma (NPC) cells by directly downregulating the expression of the alpha-1 subunit of AMPK. The inhibition of AMPK signaling in NPC cells infected with EBV was observed to increase mTOR activation and therefore increase phosphorylation of S6 kinase (S6K1) beta-1, which induces HIF-1α translocation into the nucleus. Thus, HIF-1α translocation increases aerobic glycolysis by upregulating the expression of the important glycolytic enzymes GLUT1, HK2, and LDH (21) (Figure 1).

Zika virus (ZIKV) (Class IV) also demonstrates a similar metabolic reprogramming by inhibiting AMPK signaling, increasing glucose uptake and glycolytic rates in human endothelial and mouse embryonic fibroblasts cells. Decreased AMPK signaling during infection increased the expression of glycolytic enzymes HK2, triosephosphate isomerase (TPI), monocarboxylate transporter 4, and GLUT1. Notably, activation of AMPK signaling using AMPKα activator GSK621 inhibited ZIKV replication in these cells (22).

Adenovirus-encoded protein E4 open reading frame 1 (E4ORF1) localizes in the nucleus to bind and activate Myc (proto-oncogene, basic helix-loop-helix transcription factor), an important transcription factor. This was shown to consequently upregulate the transcriptional expression of glycolytic enzymes HK2 and PFK1 in nontumorigenic breast cells and in turn elevate glucose consumption and lactate production rates while reducing oxygen consumption (showing less dependence on OXPHOS and a switch to aerobic glycolysis, i.e., the Warburg effect). The impact of this Myc translocation was found to be independent of PI3K/AKT signaling (23). Likewise, murine norovirus (MNV) (Class IV) infection was shown to increase central carbon flux (glycolysis, the pentose phosphate pathway, and the TCA cycle) in murine macrophages (RAW cells) as a result of increased AKT signaling. Inhibition of AKT phosphorylation with MK2206 reduces MNV-1 infection in RAW cells. However, no difference in phosphorylation of AMPK was found in MNV-infected RAW cells, showing an AKT-dependent mechanism (24).

However, viruses such as avian reovirus (ARV) (Class VI) facilitate replication by upregulating the mammalian target of rapamycin complex 1 (mTORC1)/eukaryotic translation initiation factor 4E (eIF4E)/HIF-1α pathway. The ARV-produced protein σA was found to upregulate HIF-1α partially through mTORC1 and eIF4E in a dehydrogenase-3 subunit beta (IDH3B) and vacuolar-type H^+^-ATPase (vATPase)-dependent manner. ARV σA protein partially activates IDH3B, which is found to phosphorylate p70S6K, downstream to mTOR. Upon activation, mTORC1 phosphorylates 4E-binding protein 1 (4E-BP1), which releases protein elF4E to facilitate translation of HIF-1α. HIF-1α further increases glycolysis by increasing the transcription of glycolytic enzymes such as HK2, PFK1, TPI, and PK. Moreover, vATPase increases the dissociation of 4E-BP1 and elF4E to increase the flux of glycolysis and the TCA cycle in its viral replication cycle (25). Interestingly, the ARV p70 protein activates AKT signaling upstream to mTORC1; however, the effect of ARV σA protein on AKT signaling still needs to be evaluated (26).

### 2.2. Glucose Transporters

Glucose is a nonpolar molecule unable to enter a cell via diffusion and thus requires GLUTs to enter a cell. GLUTs are classified by affinity, structure, and functions into different classes such as GLUT1, GLUT2, GLUT3, GLUT4, and sodium/glucose cotransporter 1 (SGLUT1). GLUT1 is expressed in all cells but is present at high levels in endothelial cells of barrier tissues such as the blood-brain barrier and human erythrocytes (27). Increased GLUT expression is often seen in viral infections. Human immunodeficiency virus (HIV) infection increases glucose transport by 300% by activating PI3K/AKT signaling, increasing GLUT1-mediated glucose uptake in T helper cells. Activation of PI3K/AKT signaling increases the phosphorylation of GLUT1 and thus its activity (28). In humans, HIV is also found to increase the expression of GLUT3 (commonly expressed in neurons of the central nervous system) in H9 lymphocytes (29). Like HIV, rhinovirus (RV) is found to mobilize extracellular glucose via PI3K signaling to increase GLUT1-mediated glucose uptake in vivo and in vitro. In glucose-deprived states, an increase in glycogenolysis was observed in RV-infected fibroblast cells to compensate for the glucose need (30). Likewise, E4ORF1 adenovirus protein enhances insulin-stimulated PI3K signaling, followed by GLUT4 translocation to the plasma membrane in adipocytes (31).

HCMV uses an early protein IE72 to replace commonly expressed GLUT1 transporters in infected human foreskin–derived fibroblast (HF) cells with more active GLUT4 transporters. IE72 modifies messenger RNA levels of the genes encoding GLUTs, downregulating GLUT1 expression and increasing GLUT4 expression. Levels of other high-affinity GLUTs also showed a modest change in expression (GLUT3, GLUT7, GLUT8, and GLUT10) and could possibly also play a role in the increase of glucose uptake. Interestingly, GLUT4 expression and translocation in HCMV-infected cells are independent of AKT signaling, unlike with HIV and RV (32).

Another example is the transmissible gastroenteritis virus (TGEV) (Class IV), a coronavirus that infects pigs and increases SGLUT1- and GLUT2-mediated glucose uptake in the epithelial cells. The glucose uptake in TGEV-infected cells is mediated via increased expression and phosphorylation of EGF receptor, which is upstream to PI3K/AKT signaling (33).

## 3. GLUTAMINOLYSIS

**Figure 2:**
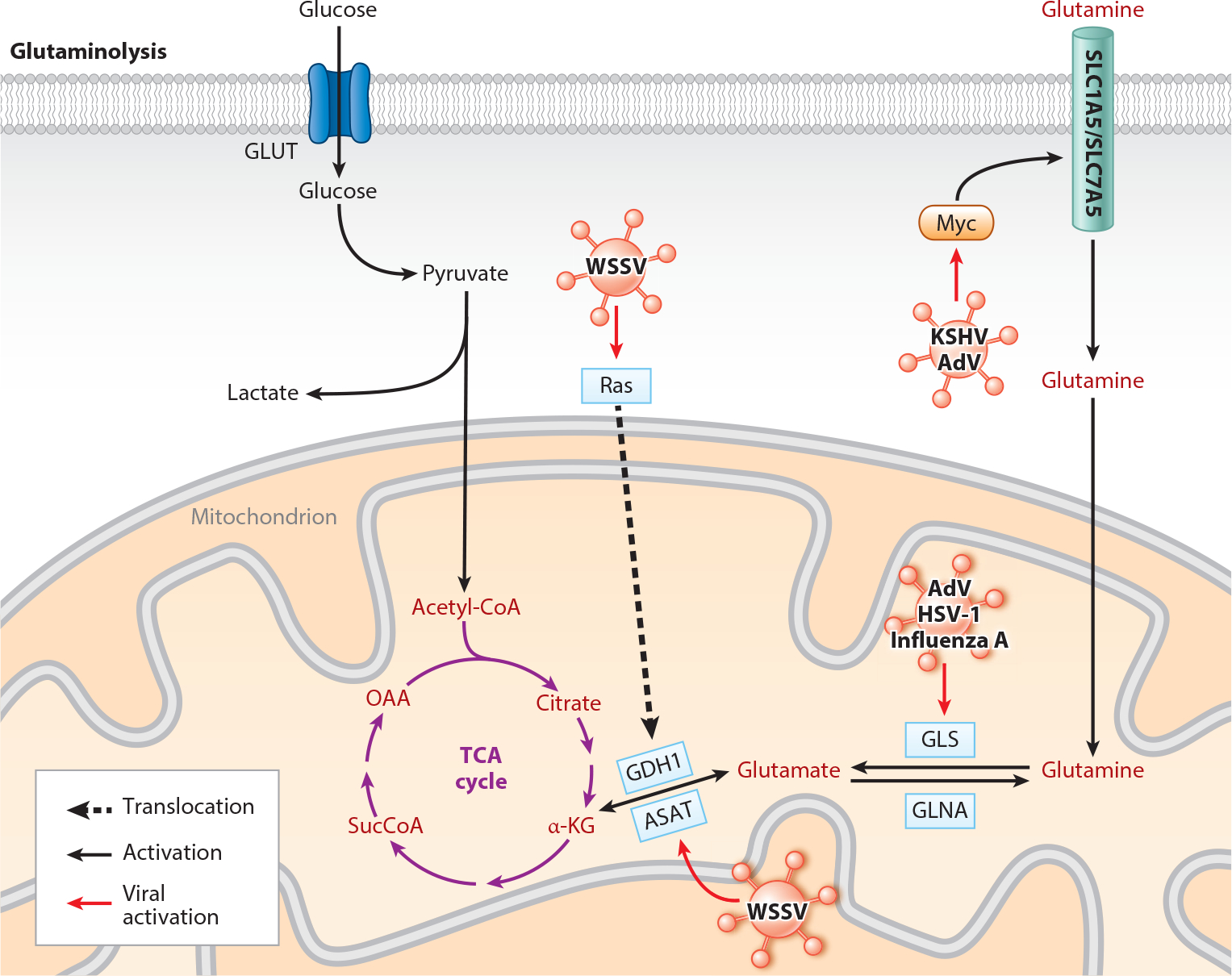
Glutaminolysis signaling and related pathways are altered by viruses. Viruses increase glutaminolysis by targeting the glutamine transporter (SLC1A5/SLC7A5) and the activity and expression of the main enzymes GLS, GDH1, and ASAT to establish infection. Red type indicates main glutamine metabolism. Abbreviations: α-KG, α-ketoglutarate; AdV, adenovirus; ASAT, aspartate aminotransferase; GDH1, glutamate dehydrogenase 1; GLNA, glutamine synthetase; GLS, glutaminase; GLUT, glucose transporter; HSV-1, herpes simplex virus 1; KSHV, Kaposi’s sarcoma herpesvirus; Myc, proto-oncogene, basic helix-loop-helix transcription factor; OAA, oxaloacetate; Ras, rat sarcoma; SucCoA, succinyl-CoA; TCA, tricarboxylic acid; WSSV, white spot syndrome virus. Created with BioRender.com.

Glutaminolysis is the process of utilization of glutamine to generate TCA cycle intermediates in the absence of pyruvate. Glutamine is converted into glutamate by glutaminase (GLS) and then converted to α-ketoglutarate by glutamate dehydrogenase (GDH), which restores the TCA cycle, allowing for the production of other intermediates such as citrate that contribute to fatty acid synthesis. Glutamine can also act as an alternative energy source, as the TCA cycle produces NADPH, which donates electrons to the ETC (Figure 2). Due to the disconnection between glycolysis and the TCA cycle seen in viral life cycles that induce aerobic glycolysis, there is a dependence on glutamine. For example, adenovirus E4ORF1 induces glutaminolysis by increasing GLS activity via Myc in infected A549 human lung carcinoma cells. Cells grown in a glutamine-free environment showed significantly decreased viral replication, indicating the need for glutamine for optimal viral survival. Adenovirus E4ORF1 was also found to activate Myc in primary human bronchial epithelial cells and to upregulate the expression of glutamine transporters SLC7A5/ASCT2 and SLC1A5/LAT1 (34). Moreover, HSV-1 (Class I) and influenza A (Class V) infection also induced mitochondrial GLS-1 activity for their optimal replication (35). The same phenomenon was seen in KSHV-infected cells, where a glutamine-free environment decreased viral replication by 75% (36). Mechanistically, KSHV was found to induce the expression of nutrient-sensing transcription factors cMyc, Max, and MondoA and its heterodimerization partner Max-like-protein X, a cellular metabolism modulator in endothelial cells. Increased expression of these transcription factors further increases the expression of glutamine transporter protein SLC1A5 and thereby increases glutaminolysis for viral survival (37).

Viral mechanisms for inducing glutaminolysis also include upregulation of the enzyme GDH, which ultimately regulates anaplerosis, converting glutamate to α-ketoglutarate. Studies with HIV subjects found they had a late immune recovery that was caused by proinflammatory molecules and glutaminolysis. Increase in L-glutamate concentration in HIV infection can be associated with microbial translocation and damage of the gastrointestinal tract (38). Glutaminolysis also affects cell replication through activation of mTORC1 (39).

In another study, shrimp infected with WSSV have revealed that the virus upregulates the expression of GDH and aspartate aminotransferase (ASAT). ASAT is another enzyme-like GDH involved in the conversion of glutamate to α-ketoglutarate. Notably, WSSV infection induces the Warburg effect in infected shrimp cells, and thus following the previous discussion, anaplerosis through glutamine is needed for viral replication. This increase in the WSSV-infected cells is regulated by Ras signaling, not by PI3K/AKT/mTOR signaling (11). Therefore, it may be helpful to target glutaminolysis as a potential therapeutic strategy in virus-infected cells.

## 4. LIPID METABOLISM

**Figure 3:**
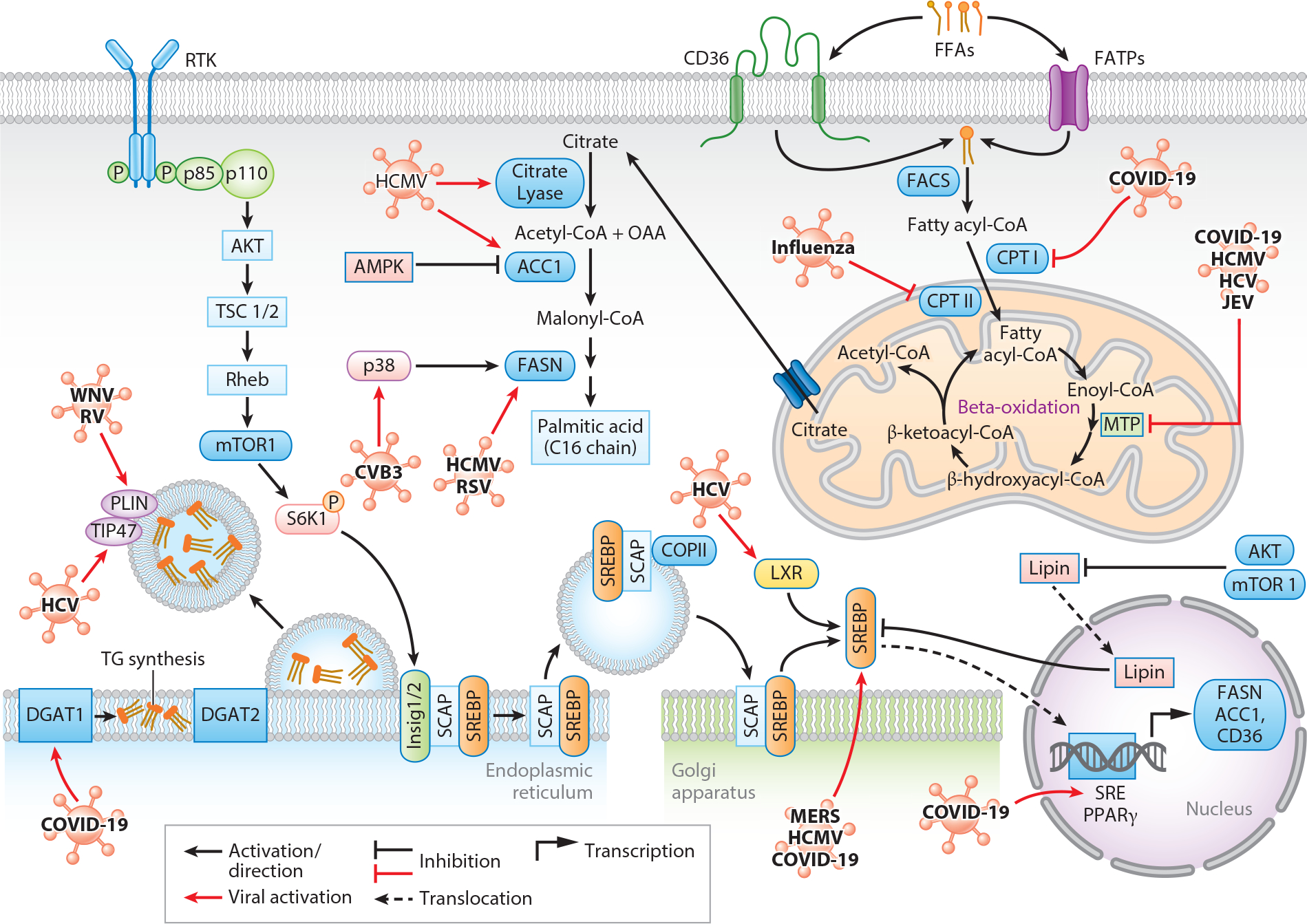
Lipid metabolism and related pathways are altered by viruses. Upon infection, there is an increase in lipid synthesis, which is mediated by increasing the activity of main fatty acid synthesis enzymes such as ACC and FASN and transcription factors such as SREBP. Moreover, this figure also represents virus modulation of beta-oxidation and lipid droplet formation. Abbreviations: ACC, acetyl-CoA carboxylase; AMPK, AMP-activated protein kinase; COPII, coat protein complex II; COVID-19, coronavirus disease 2019; CPT, carnitine palmitoyltransferase; CVB3, coxsackievirus B3; DGAT, diacylglycerol O-acyltransferase; FACS, fatty acyl-CoA synthetase; FASN, fatty acid synthase; FATP, fatty acid transport protein; FFA, free fatty acid; HCMV, human cytomegalovirus; HCV, hepatitis C virus; JEV, Japanese encephalitis virus; LXR, liver X receptor; MERS, Middle East respiratory syndrome; mTOR, mechanistic target of rapamycin; MTP, mitochondrial trifunctional protein; OAA, oxaloacetate; P, phosphate group; PLIN, perlipin; PPARγ, peroxisome proliferator-activated receptor γ; Rheb, Ras homolog enriched in brain; RSV, respiratory syncytial virus; RTK, receptor tyrosine kinase; RV, rhinovirus; S6K1, S6 kinase beta-1; SCAP, SREBP cleavage-activating protein; SRE, sterol regulatory element; SREBP, sterol regulatory element-binding protein; TG, triacylglycerol; TIP47, tail-interacting protein-47; TSC, tuberous sclerosis complex; WNV, West Nile virus. Created with BioRender.com.

Lipid metabolism involves pathways related to either storage or breakdown of lipids, including fatty acids. Fatty acid synthesis usually occurs in the liver and adipose tissues. The critical step of fatty acid synthesis starts with acetyl-CoA being converted to malonyl-CoA by carboxylation with the help of acetyl-CoA carboxylase (ACC), the most regulated enzyme in this process (40) (Figure 3). However, fatty acid oxidation is the process of fatty acid breaking down into acetyl-CoA to produce ATP. It begins with the fatty acids being transferred from the cytoplasm (where fatty acid synthesis occurs) to the mitochondria. The essential step, known as beta-oxidation, occurs in mainly four stages: oxidation, hydration, oxidation again, and thiolase activity, which are correspondingly activated by acyl-CoA dehydrogenase, enoyl-CoA hydratase, hydroxyacyl-CoA dehydrogenase (HADH), and ketoacyl-CoA thiolase (41). Fatty acid metabolism has been found to be associated with glycolysis in many ways. Pyruvate, a product of glycolysis, is also an essential component of fatty acid synthesis. Furthermore, the rate of fatty acid oxidation relies on the availability of glucose stores (42).

There are several vital factors regulating fatty acid metabolism. One of the proteins, mTOR, controls fatty acid synthesis, adipogenesis, esterification, and hydrolysis of triacylglycerols (TAGs)/lipolysis. Other vital factors that partake in fatty acid metabolism are sterol regulatory element-binding proteins (SREBPs). SREBPs are a class of transcription factors that bind sterol regulatory elements (SREs) in DNA. The SREs are present at the promoters of genes involved in lipid synthesis, such as ACC1, fatty acid synthase (FASN), sterol CoA desaturase-1, and lipoprotein lipase (43). SREBP-1, which can be spliced into SREBP-1a and SREBP-1c upon activation, is involved with lipids. SREBP-1a is engaged in global lipid synthesis and growth, and SREBP-1c is involved in fatty acid synthesis and energy storage (44). SREBP-2 is mainly involved in cholesterol regulation but has indirect abilities to upregulate lipid biosynthesis (45).

SREBP cleavage-activating protein (SCAP) forms a complex with SREBP on endoplasmic reticulum (ER) membrane and helps transport of SREBP from the site of synthesis (ER) into the site of its cleavage (Golgi). SCAP has a sterol binding domain that regulates the synthesis and lipid uptake within cells (46). In surplus of sterols, insig-1/2 binds the SCAP complex on the ER membrane that, when bound, blocks coat protein complex II (COPII)-mediated movement of the SREBP-SCAP complex to the Golgi membrane (47). In this process, an ER membrane–bound, GTP binding protein, Sar-1, attracts the cytosolic protein Sec23/24, which enhances binding of coat proteins to the budding vesicle. These COPII vesicles lead to the movement of the SREBP-SCAP complex to the targeting motif on the Golgi protein. At the Golgi, two membrane-bound proteases (S1P and S2P) recognize SREBP and cleave it, freeing it from the membrane and translocating it to the nucleus, thus increasing the transcription of lipogenic genes (ACC and FASN) (48, 49). Another critical pathway that leads to the activation of SREBPs is activation of AKT and mTORC. Activation of mTORC phosphorylates S6K1 and increases SREBP-1c translocation to the nucleus by dissociating the SCAP-SREBP complex (50). AKT inhibits a protein called lipin, which sequesters SREBP in the nucleus. Here, mTORC plays a major role by hyper-phosphorylation of lipin, which prevents it from going into the nucleus and hence increasing SREBP translocation (51).

### 4.1. Viral Manipulation of Fatty Acid Synthesis

HCMV-infected HF cells show a substantial increase in ACC1, ATP-citrate lyase, and FASN, all rate-limiting enzymes for lipid biosynthesis. In HF cells, after 24 h of HCMV infection, mature SREBP-1 was detected in the cell’s nucleus, resulting in fatty acid synthesis and the accumulation of lipid droplets (LDs) in the cytoplasm, which assist in the replication and production of the lipid bilayer required by the dsDNA HCMV (52). Another study showed that in HCMV-infected MRC-5 fibroblasts, there was the potential presence of SREBP-2 in the nucleus. The mechanism of proteolytic activation of SREBP-2 leads to an increase in ACC1, a key driver for lipid synthesis (53). Middle East respiratory syndrome coronavirus (Class IV) also increases lipogenesis by utilizing the SREBP-1 pathway to increase its replication. Inhibition of the SREBP-1 pathway with betulin acts as an antiviral intervention (54).

Latent membrane protein-1 is the major transforming protein of EBV that induces FASN gene expression and increases LD formation in B cells. However, the increase in the expression of FASN is not through SREBPs, and there is actually an increase of ubiquitin-specific peptidase 2a, a ubiquitin-specific protease that prevents degradation of FASN by proteasomes and hence stabilizes FASN to increase lipogenesis (55).

Coxsackievirus B3 (CVB3) (Class IV) infection also induces the expression of FASN in human lymphoblast cells (Raji cells) by p38 mitogen-activated protein kinase (MAPK). Inhibitors of MAPK and FASN are shown to reduce CVB3 replication in these cells as well (56). Respiratory syncytial virus (RSV) (Class V)-infected human lung epithelial cells (A549) were also found to induce FASN. TVB-3166, an FASN inhibitor, reduces the viral replication of RSV-A and RSV-B and lowers their infectivity in A549 cells as well as in RSV-A-infected mice. The cellular signaling leading to FASN induction in RSV infection is still not explicit (57).

HCV utilizes saturated and monounsaturated fatty acids for their replication. Inhibition of fatty acid synthesis by inhibiting ACC and FASN results in a decrease in HCV replication in Huh-7 cells. However, LXR-SREBP-1 signaling was not found to play any role in the replication of HCV. Geranylgeranylation, a universal lipid post-translational modification, is also involved in this interaction because its inhibition also suppresses viral replication (58).

As an exception, porcine reproductive and respiratory syndrome virus (PRRSV) (Class IV) increases AMPK phosphorylation, and that increases the phosphorylation of ACC1, making it inactive in PRRSV receptor CD163 stably expressed kidney cell lines. However, inhibition of FASN inhibits PRRSV replication, which indicates that PRRSV uses fatty acids for its replication, but the cellular signaling pathway to increase fatty acid synthesis is different from the AMPK-ACC pathway (59).

### 4.2. Viruses Alter Lipid Droplet Formation

LDs are ubiquitous organelles that contain neutral lipids within their core and are surrounded by a phospholipid monolayer (60). They can release TAGs through lipolysis upon activation, typically during cell growth or nutrient depreciation (61). Critical regulators of LD formation include diacylglycerol O-acyltransferase 1 and 2 (DGAT1 and DGAT2). These enzymes bind to the ER membrane and regulate TAG synthesis and packaging within the phospholipid bilayer to form LD budding, which is then released into the cytoplasm (62). LDs can be formed through the activation of SREBPs. In particular, SREBP-1 activation leads to the production of lipogenic enzymes and produces perilipin (PLIN), an LD membrane surface protein associated with the production of LDs (63) (Figure 3).

Viruses alter LD formation by taking advantage of the LD components including triglycerides, cholesterol esters, and the highly dynamic membrane trafficking system in order to enhance their replication cycle (64). For example, in SARS-CoV-2-infected primary human monocytes, human lung epithelial cell lines (A549), and human lung microvascular endothelial cell lines, there is an increase of LDs in the cytoplasm due to upregulation of SREBP-1 and peroxisome proliferator-activated receptor-γ. These two transcription factors play a critical role in upregulating lipogenic enzymes and fatty acid transporters (CD36), respectively. SARS-CoV-2 also modulates DGAT-1, a key regulator in TAG synthesis and LD formation to increase the protein trafficking to LDs that increase lipid biogenesis. Inhibition of DGAT-1 by A922500 reduces the SARS-COV-2-induced cell death and inflammation in primary monocytes (65).

HCV replicates and produces structural and nonstructural (NS) proteins. In HCV-infected human liver cancer–derived Huh7 cells, a portion of NS5A, a key regulator of the HCV life cycle (66), localizes to LDs. NS5A carries newly synthesized viral RNA to LDs for virion assembly through protein-protein interactions with tail-interacting protein-47 (TIP47). TIP47 binds to the LD surface and is critical for LD regulation. TIP47 interacts with NS5A on LDs and NS5A-containing replication complexes at the ER membrane, thus increasing HCV RNA replication by making LD accessible for either the creation of the viral membrane or integration of LDs into the membrane (67). Another virus utilizing host machinery and LDs for viral replication is ZIKV, which interrupts cell signaling networks by expressing different structural and NS viral proteins, which exhibit distinctive subcellular distribution in host cells. The structural protein capsid was found to localize in nucleoli, Golgi apparatus, and LD of host cells, and NS4B has been known to alter the lipid composition of infected cells in humans, mainly through sphingolipids; however, the function associated with the interaction of different organelles still needs to be determined (68). Dengue virus (DENV) also takes advantage of LDs during viral assembly. In DENV-infected baby hamster kidney (BHK) cells, the hydrophobic a2 helix of the viral capsid protein attaches to the phospholipid monolayer of LD, which is critical for virus replication (69). Utilizing a similar approach as HCV, West Nile virus (WNV) capsid proteins interact with LDs as seen in BHK-21 cells. The most probable mechanism for this connection is the association of WNV capsid protein binding with PLIN3, a protein essential for the mobilization of fat that is essential for WNV replication (70). Rotaviruses also utilize LDs through their NS proteins NSP2 and NSP5 and their structural proteins VP1, VP2, VP6, and perilipin membrane surface proteins. In rotavirus-infected embryonic Rhesus monkey kidney tissue cells (MA-104), NSP5 localizes around LDs. Rotaviruses interact with PLIN1 and PLIN2 to recruit LD components to the viral site of replication and the viroplasms (71). Influenza viruses utilize LDs through ER stress and autophagy. Cells with ER stress accumulate lipids, and influenza virus utilizes this to generate viral replication through the host machinery. Influenza virus downregulates lipid-associated and -modulating proteins ω−3 poly-unsaturated fatty acid-derived lipid mediator protectin D1 and interferon γ inducible (viperin) to established infection. Increases in LDs induced ER stress and reactive oxygen species (ROS) in host cells. Inhibition of LD formation using atorvastatin in Madin-Darby canine kidney cells reduces influenza virus replication by 90% to 95% as compared to ER stress, autophagy, and ROS inhibitors. This indicates that targeting the LD machinery can be useful in generation of potential therapeutics for viral infection (72).

### 4.3. Viral Manipulation of Beta-Oxidation

Fatty acid oxidation is the process of breaking down fatty acids into acetyl-CoA and eventually into ATP (Figure 3). Long-chain fatty acids must be taken to the mitochondria or into peroxisomes to be oxidized. The critical enzyme on the outer mitochondrial membrane responsible for transporting fatty acids into the mitochondria is carnitine palmitoyltransferase I (CPT I). ATP is generated using fatty acids by the process of beta-oxidation, which happens within mitochondria and peroxisomes (41).

Because beta-oxidation is a significant energy source for various systems in times of high demand, viruses have developed methods to override critical cell signaling pathways to upregulate/downregulate beta-oxidation. Viruses downregulate beta-oxidation, leading to a surplus of free fatty acids in the cytoplasm for viral replication (73). However, viruses may upregulate beta-oxidation to increase ATP production for efficient viral replication. Influenza virus-infected human patient fibroblasts display a significant increase in heat stress, which leads to the downregulation of CPT II, the inner mitochondrial transferase responsible for bringing free fatty acids into the mitochondria (74). SARS-CoV-2 has been shown to impair fatty acid oxidation in alveolar epithelial cells isolated from mouse models of acute pulmonary endotoxemia–induced damage. In these infected cells, there is a decrease in the expression of PPARG1c and CPT1a, which regulate mitochondrial function and CPT formation, respectively (75). HCV NS5A in Huh7 cells interacts with the mitochondrial trifunctional protein (MTP). MTP is involved in hydration, oxidation, and thiolysis, the final three steps of beta-oxidation. NS5A is hypothesized to interact with MTP through protein-protein interactions, leading to the stability of MTP and downregulation of beta-oxidation (76). HCMV also blocks the function of the MTP through HCMV-produced viral mitochondria-localized inhibitor of apoptosis (vMIA) protein, which binds to viperin. vMIA translocates viperin to the mitochondria, where it interacts with MTP and downregulates beta-oxidation in HCMV-infected human fibroblasts (77). One of the NS proteins of the Japanese encephalitis virus (JEV), NS5, also interacts with the HADH trifunctional multienzyme complex subunit alpha and subunit beta, two MTP proteins, to block beta-oxidation in JEV-infected human alveolar basal epithelial cells (A549). NS5-M19A mutation was not able to block beta-oxidation and reduce inflammation in host cells (78).

However, DENV upregulates beta-oxidation. Inhibition of beta-oxidation using Etomoxir (CPT1 inhibitor) decreases viral production, indicating the importance of beta-oxidation in viral replication. However, the mechanism of DENV-induced beta-oxidation remains unclear (79). Vaccinia virus was also shown to upregulate beta-oxidation in green monkey BSC40 cells to manipulate cells to produce more ATP for viral replication and protein synthesis (80).

## 5. A NOVEL MECHANISM TO MANIPULATE HOST METABOLISM: DISCOVERY OF VIRAL INSULIN AND INSULIN-LIKE GROWTH FACTOR-LIKE PEPTIDES

In the previous sections, we reviewed the mechanisms in which viruses affect host metabolism for their benefits. However, we recently discovered a totally novel mechanism in which viruses directly mimic host hormones to alter metabolic pathways. One of these strategies is based on viral mimicry, in which viruses produce peptides similar to host factors that have crucial roles in regulating the immune response or cellular growth (81–83). As we recently reviewed (84), viral mimicry can occur by divergent or convergent evolution. In divergent evolution, a gene is transferred from a host genome to a viral genome (85), and the viral mimic has significant similarity to the host protein. However, in convergent evolution, random genetic mutations of viral sequences create factors that mimic a host protein’s function (86, 87). These viral mimics have limited sequence similarity with the host proteins; however, they have functional similarities.

Peptide hormones play a central role in regulating metabolism, growth, and development. In our first study, we hypothesized that viruses mimic mammalian peptide hormones to regulate host functions. To test this, we performed a comprehensive bioinformatics search in all available viral genomes. Using a local BlastP library, we searched for the presence of peptide sequences with high sequence similarity to 62 human peptide hormones and regulatory proteins (88). We identified viral sequences that showed significant similarity with 16 different human peptide hormones, including insulin, insulin-like growth factors 1 and 2 (IGF-1 and IGF-2), tumor necrosis factor, endothelin-1 and −2, interleukin 6, and adiponectin (88, 89). Until we discovered viral hormones, viral mimicry examples were limited to immunomodulatory proteins and growth factors (90–95). The discovery of viral hormones opened a new avenue in understanding host-pathogen interactions and indicated a new mechanism of viral pathogenesis.

Because of the significant sequence similarity, we focused on the characterization of viral insulin/IGF-like peptides (VILPs). VILPs were identified in four viruses that belong to the *Iridoviridae* family. These viruses were initially isolated from fish (92, 96–98). However, we and others identified the sequences of these viruses in human enteric virome and plasma samples (87, 99–103). Amino acid sequences of VILPs are 30–50% identical to human insulin/IGF-1. Notably, all six critical cysteine residues that form intrachain and interchain disulfide bonds in the insulin/IGF tertiary structures were conserved among the four VILPs. Insulin is a double-chain (dc) peptide (consisting of an A-chain and B-chain that are linked together by disulfide bonds), and IGFs are single-chain (sc) peptides (containing an additional C domain that connects the A- and B-chains). Thus, we chemically synthesized the VILPs in both their insulin-like (i.e., dc) and IGF-like (i.e., sc) forms. In our first study (88), we characterized sc forms of grouper iridovirus (GIV), Singapore grouper iridovirus (SGIV), and lymphocystis disease virus-1 (LCDV-1) VILPs. We showed that scVILPs are weak ligands of human insulin receptor (IR) but potent ligands of IGF-1 receptor (IGF1R) in vitro. VILPs can bind to human insulin and IGF1Rs and stimulate downstream insulin/IGF signaling, both PI3K/AKT and Ras/MAPK pathways. Furthermore, scVILPs can also increase glucose uptake in adipocytes and stimulate the proliferation of fibroblasts. An insulin tolerance test demonstrated that LCDV-1 scVILP could significantly lower the blood glucose of mice in high concentrations. These results taken together demonstrated that VILPs are active members of the insulin superfamily.

In our recent study, we focused on the characterization of chemically synthesized dc versions of GIV, SGIV, and LCDV-1 VILPs (104). We showed that GIV and SGIV dcVILPs bind and stimulate both human IR and IGF-1R in vitro and in vivo. To better understand in vivo characteristics of the VILPs, we designed an in vivo infusion experiment. We showed that GIV dcVILP stimulated significantly higher (approximately two fold) glucose uptake in white adipose tissue (WAT) compared to insulin. However, no differences were observed in the other insulin-sensitive tissues (skeletal muscle, brown adipose tissue, and heart) (104). This effect in WAT was associated with higher AKT phosphorylation and GLUT4 gene expression upon GIV dcVILP stimulation compared to insulin. This result suggests that GIV dcVILP has some characteristics that specifically target WAT.

In addition to these agonistic effects, we observed a unique feature of LCDV-1 scVILP. We showed that LCDV-1 scVILP has antagonistic properties on IGF-1R (105), as the activation of the IGF-1R by IGF-1 is strongly repressed by LCDV-1 scVILP. This effect is IGF-1R specific and does not affect IR. Functionally, we showed that LCDV-1 scVILP inhibits IGF-1-stimulated migration of cervical cancer cells. These results taken together demonstrated that VILPs are new members of the insulin superfamily with some unique characteristics that are potentially advantageous for the viral infection or replication cycle. Characterization of VILPs as novel ligands of insulin and IGF1Rs has already improved our knowledge of insulin/IGF-1 action; however, their role in host-pathogen interactions is not known. The only report of VILP activity in host-pathogen interactions is the overexpression of SGIV VILP in grouper cells (106). The researchers showed that SGIV VILP overexpression promoted grouper embryonic cells’ growth by stimulating G1/S phase transition. It was also proven that SGIV VILP facilitated SGIV replication in grouper cells.

**Figure 4:**
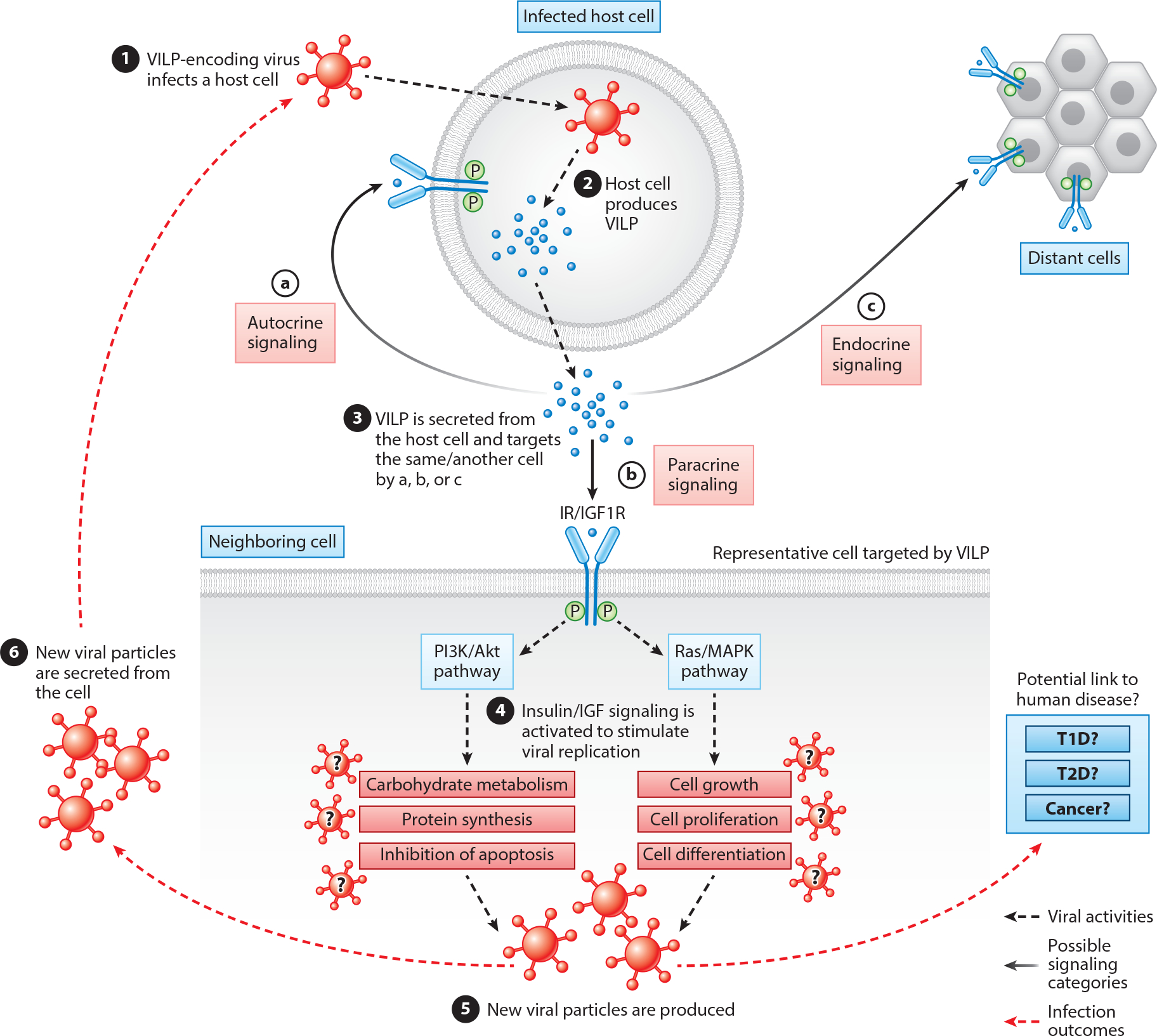
Potential effects of VILPs on the host cell metabolism. We hypothesize that upon infection, VILPs will be translated and secreted by the host cells and act on a target cell in an autocrine (*a*), paracrine (*b*), or endocrine (*c*) manner. In the target cells, VILP may activate several aspects of insulin/IGF signaling and alter several pathways regulating carbohydrate metabolism, cell growth, proliferation, and apoptosis. Abbreviations: IGF, insulin-like growth factor; IGF1R, insulin-like growth factor 1 receptor; IR, insulin receptor; MAPK, mitogen-activated protein kinase; P, phosphate group; PI3K, phosphoinositide 3-kinase; Ras, rat sarcoma; T1D/T2D, type 1 diabetes/type 2 diabetes; VILP, viral insulin/IGF-like peptide. Created with BioRender.com.

We anticipate that VILP-carrying viruses target insulin/IGF-1 signaling to manipulate the glucose metabolism and cell cycle upon infection to promote their replication (Figure 4). Furthermore, insulin and IGF-1 are mitogenic (107, 108) and antiapoptotic factors (109, 110), potentially helping the virus replicate easily while protecting the cell to initiate apoptosis. We still do not know whether mammalian and human cells are susceptible (permit entry of the virus) and permissive (support reproduction of the virus) for VILP-carrying viruses. Although members of the *Iridoviridae* family are known to infect a diverse array of cold-blooded vertebrate hosts, there are few studies examining their potential to infect mammalian cells. GIV and SGIV belong to the *Ranavirus* genus of the *Iridoviridae* family, and some related ranaviruses can enter mammalian cells (111–115). For example, invertebrate iridescent virus 6 was shown to infect murine fibroblast cells and stimulate an antiviral innate immune response through the RIG-I-like receptor pathway in mammalian cells (113). In addition, frog virus 3 (FV3), another ranavirus like GIV and SGIV, was able to express some early genes in Chinese hamster ovary cells and triggered cell death (115). Moreover, FV3 infects rat Kupffer cells, and FV3 viral particles were also observed in phagocytic vacuoles and endocytic compartments (111). Ranavirus researchers suggest that macrophages are the integral targets for ranavirus infections (111–114), indicating a universal underlying mechanism governing the ranavirus entry to cells from evolutionarily distant organisms (112). If VILP-carrying viruses can infect human cells, they might be linked to human diseases, including type 1 diabetes based on molecular mimicry mechanism, type 2 diabetes based on insulin signaling impairment, and cancer with VILP’s mitogenic effects (84).

It is important to note that our knowledge of VILP-carrying viruses is quite limited. There are only 10,462 viral genomes available on the NCBI databases (NIH, as of February 10, 2021), while a minimum of 320,000 mammalian viruses are predicted to exist, and most of them are waiting for discovery (116). We identified four VILP-carrying viruses in the available viral genomes and expect to make future discovery of tens of other viral species that carry VILPs.

## 6. CONCLUSION AND FUTURE DIRECTIONS

Viral regulation of host metabolism is an essential component of viral replication and survival. Because the number of completed studies is quite limited in this field, it is not possible to make a concrete statement whether some specific classes of viruses are targeting specific pathways. As explained in detail, the viruses target various signaling pathways including P13K/AMPK/mTOR signaling to manipulate glucose metabolism independent of viral classes and their hosts. Viruses also activate transcription factors such as Myc, SREBP, and HIF-1α to induce its replication and survival in host cells. Inhibition of these signaling pathways and several metabolic enzymes was shown to decrease viral replication in vitro or in vivo. Furthermore, the discovery and characterization of VILPs open a new avenue in host-pathogen interactions in which viruses have the ability to mimic the hormones that directly regulate host metabolism. A better understanding of viral mechanisms targeting host metabolism will help us to better understand viral pathogenesis and has the potential to open a new avenue in designing novel antiviral therapies. Our knowledge of these signaling pathways altered by viruses and their effects on the host is still very limited and needs to be explored further.

## Supplementary Material

Supplemental Table 1
