# Supplementary material for: Viruses and Metabolism: The Effects of Viral Infections and Viral Insulins on Host Metabolism
Source: Annu Rev Virol. Author manuscript; Available in PMC 2022 Jun 8. (PMC9175272; doi:10.1146/annurev-virology-091919-102416)
Supplement: Supplemental Table 1 [file NIHMS1807389-supplement-Supplemental_Table_1.pdf]

Supplementary Table 1: Represents the target signaling proteins and enzymes by viruses in Glucose, glutamine and lipid metabolism.

S.No.	Glucose Metabolism					
	**Family of Viruses**	**Baltimore Class**	**HOST**	**Virus**	**Mechanism**	Ref.
1	Coronaviridae	ssRNA (+) IV	Human	SARS-CoV-2	Disrupt mitochondrial function and increase glycolytic rate	16
2	Herpesviridae	dsDNA (+/-) I	HUMAN	Epstein Barr Virus (EBV)	Encodes miRNA((EBV-miR-Bart1-5P) decrease AMPK	21
3	Flaviviridae	ssRNA (+) IV	Human and non-human premates	Zika virus	Reduce AMPK phosphorylation increase glucose uptake	22
4	Caliciviridae	ssRNA (+) IV	Mouse	Murine Norovirus (MNV)	Upregulating AKT signaling	23
5	Adenoviridae	dsDNAv(+/-) I	Human	Adenovirus	Adenovirus E4ORF1 binds to Myc to increase glycolytic enzyme transcription. Also increase PI3K to increase GLUT 4 translocationE4	24,32
6	Retrovirdiae	ssRNA (+) VI	Chicken	Avian reovirus (ARV)	Upregulates mTORC1/elF4E/HIF1 a to enhance the expression of HK2, PFK, TPI and PK	25,26
7	Iridoviridae	dsDNAv(+/-) I	fish	Infectious Spleen and Kidney Necrosis Virus (ISKNV)	Promotes the expression of HK1, LDH, G6PDH	17
8	Picornaviridae	ssRNA (+) IV	Human	Rhinovirus (RV)	Activate the PI3K pathway increase phosphorylation of GLUT 1. Also increase glycogenolysis	30
9	Retrovirdiae	ssRNA (+) VI	Human	HIV	Upregulate PI3K/AKT signaling to increase GLUT1 phosphorylation. Also increase GLUT 3 expression	29
10	Herpesviridae	dsDNAv(+/-) I	Pigs	Human Cytomegaloviru s (HCMV)	Replace GLUT1 and increase the expression of GLUT4 to increase glycolysis.	33

11	Coronaviridae	ssRNA (+) IV	Pig	Transmissible Gastroenteritis Virus (TGEV)	Increase EGFR phosphorylation and thereby phosphorylation of SGLUT1 and GLUT2	31
**Glutaminolysis**						
1	Adenoviridae	ssRNA (+) IV	Human	Adenovirus	Adenovirus E4ORF1 binds to Myc increase GLS1 activity	34
2	Herpesviruses	dsDNA (+/-) I	Human	Herpes simplex virus (HSV)	Increase GLS activity	35
3	Orthomyxoviridae	dsDNAv(+/-) I	Human and non-human premates	Influenza virus	Increase GLS activity	35
4	Herpesviruses	ssRNA (+) IV	Human	Kaposi's sarcoma-associated herpesvirus (KSHV)	Increase interaction of Myc-Max-MondoA and which increases Glutamine transporter SCL1A5 expression	35,36
5	Retrovirdiae	dsDNAv(+/-) I	Human	HIV	Increase L-glutamine concentration, no mechanism known	38
6	Nimaviridae	ssRNA (+) IV	Shrimp	White spot syndrome virus (WSSV)	Increase Ras signaling and thereby increase expression of GDH and ASAT	11
**Fatty Acid Synthesis**						
1	Herpesviridae	dsDNAv(+/-) I	Human/Monkey	Human Cytomegalovirus (HCMV)	Increase SREBP-1 and SREBP-2 translocation to increase lipogenesis	52
2	Coronaviridae	ssRNA (+) IV	Human	Middle East respiratory syndrome coronavirus (MERS-CoV)	Increase translocation of SREBP-1 to nucleus and hence increase fatty acid synthesis	53
3	Herpesviridae	dsDNAv(+/-) I	Human	Epstein Barr Virus (EBV)	Increase USP2a expression which prevents FASN degradation	54
4	Picornavirus	ssRNA (+) IV	Human	Coxsackievirus	Increase p38 MAPK activity have a role in regulation of FAS	55
5	Pneumoviridae	ssRNA (-) V	Human	Respiratory syncytial virus (RSV)	Increase expression of FASN	56
6	Flaviviridae	ssRNA (+) IV	Human	Hepatitis C virus (HCV)	Increase ACC/ FASN expression. Induction of lipogenic gene expression results in increase of geranylgeraniol stimulate HCV RNA replication.	57

7	Arteriviridae	ssRNA (+) IV	Pig	Porcine reproductive and respiratory syndrome virus (PRRSV)	Increase AMPK phosphorylation and thereby ACC. But replication is dependent of FASN activity	58
**Lipid Formation**						
1	Coronaviridae	ssRNA (+) IV	Human	SARS-CoV-2	Increase expression of SREBP-1 and PPARG which increase CD36 expression. There is also modulation od DGAT-1 which increase protein trafficking to LDs.	64
2	Flaviviridae	ssRNA (+) IV	Human	Hepatitis C virus (HCV)	NS5A interact with TIP47 making LDs accessible for membrane formation	65
3	Flaviviridae	ssRNA (+) IV	Human and non-human premates	Zika virus	Alter composition of lipid in different compartment of cells	66
4	Flaviviridae	ssRNA (+) IV	Human	Dengue Virus	Attached hydrophobic residue a2 on phospholipid monolayer.	67
5	Flaviviridae	ssRNA (+) IV	Human	West Nile virus (WNV)	Capsid protein interacts with PLIN3 essential for fat mobilization	68
6	Reoviridae	ssRNA (+) VI	Human	Rotavirus	Interacts with PLIN1 and PLIN2 to recruit LDs component to viral site	69
7	Orthomyxoviridae	ssRNA (-) V	Human and non-human premates	Influenza virus	Downregulate PD-1 and viperin to recruit LDs.	70
**Beta-oxidation**						
1	Orthomyxoviridae	ssRNA (-) V	Human and non-human premates	Influenza virus	Decrease expression of CPTII	72
2	Coronaviridae	ssRNA (+) IV	Human	SARS-CoV-2	Decrease expression of CPTI and PPARG1c	73
3	Flaviviridae	ssRNA (+) IV	Human	Hepatitis C virus (HCV)	NS5A interacts with MTP to downregulate beta oxidation	74
4	Herpesviridae	dsDNAv(+/-) I	Human	Human Cytomegalovirus (HCMV)	Block MTP through vMIA to downregulate beta oxidation via viperin	75
5	Flaviviridae	ssRNA (+) IV	Human	Japanese encephalitis Virus	Interacts with MTP to downregulate beta-oxidation	76
6	Flaviviridae	ssRNA (+) IV	Human	Dengue Virus	Upregulate beta-oxidation	77

7	Poxviridae	dsDNA (+/-) I	Human	Vaccinia virus	Upregulate beta-oxidation	78
